# How far is too far? Geographic distance and outcomes in acute ischemic stroke in a middle-income country

**DOI:** 10.3389/fneur.2026.1816127

**Published:** 2026-04-09

**Authors:** Tony Fabian Alvárez, Alejandra Mendoza-Monsalve, Edgar Fabián Manrique-Hernandez, Maria Fernanda García Diaz, Leidy Dayanna Jaimes Figueroa, Alexandra Hurtado-Ortiz, Maricel Licht-Ardila, Federico Silva

**Affiliations:** 1 Center of Excellence for Stroke Care, Neurological Institute. Hospital Internacional de Colombia HIC- Fundación Cardiovascular de Colombia FCV- Fundación Universitaria FCV, Piedecuesta, Santander, Colombia; 2 Epidemiology Department, Hospital Internacional de Colombia HIC- Fundación Cardiovascular de Colombia FCV- Fundación Universitaria FCV, Piedecuesta, Santander, Colombia

**Keywords:** geographic locations, health services accessibility, ischemic stroke, mortality, treatment outcome

## Abstract

**Introduction:**

Geographic distance to specialized centers is a critical barrier to stroke care, influencing access, treatment delays, and early clinical outcomes.

**Objective:**

To evaluate the association between geographic distance to a Comprehensive Stroke Center (CSC) and clinical outcomes in patients with acute ischemic stroke (AIS) in a middle-income country.

**Methods:**

A Retrospective cohort study using a prospective registry of adult AIS patients in Colombia. Exposure was road distance (km) from the municipality of origin to the CSC. Outcomes included in-hospital case-fatality and functional status at discharge (modified Rankin Scale [mRS]). Multivariable logistic and ordinal regressions adjusted for age, neurological severity (NIHSS), and mode of arrival.

**Results:**

Of 529 patients, 40.64% lived ≤12.8 km and 24.76% lived ≥118 km from the CSC. Increased distance was associated with higher interfacility referrals (85.50% vs. 51.16%, *p* < 0.001). Intrahospital quality metrics and in-hospital case-fatality (*p* = 0.371) were comparable across distance groups. In multivariable analysis, in-hospital case-fatality was driven by older age (OR 1.03; 95% CI 1.00–1.06) and higher NIHSS (OR 1.15; 95% CI 1.11–1.197), while mode of arrival was also independently associated with mortality and sex was not independently associated. Geographic distance was independently associated with worse functional outcomes at discharge (OR 1.25; 95% CI: 1.09–1.44 per ln[km] increase), alongside age (OR 1.03; 95% CI: 1.02–1.04).

**Conclusion:**

Greater geographic distance was independently associated with worse functional outcomes at discharge. However, distance did not adversely affect in-hospital quality of care or in-hospital case-fatality. These findings highlight the need to strengthen prehospital systems and post-acute rehabilitation to reduce geography-related disparities in functional recovery.

## 1 Introduction

Stroke remains one of the leading causes of mortality and the foremost cause of long-term physical and cognitive disability among adults worldwide (1). Ischemic stroke accounts for the majority of cases and contributes substantially to stroke-related disability, healthcare utilization, and socioeconomic burden. In recent decades, the global burden of stroke has shifted toward low- and middle-income countries, where population aging, fragmented health systems, and limited access to specialized care are associated with poorer outcomes compared with high-income settings (2).

Timely reperfusion therapies, namely intravenous thrombolysis (IVT) and mechanical thrombectomy (MT), have consistently demonstrated substantial benefits in reducing disability and improving functional outcomes in patients with acute ischemic stroke (3, 4). The widely accepted principle that “time is brain” underscores the rapid progression of irreversible ischemic injury, with an estimated loss of approximately 1.9 million neurons per minute of untreated large-vessel occlusion (5). Both randomized trials and real-world registries have shown that delays in reperfusion are strongly associated with higher mortality and worse functional outcomes at 90 days (5, 6).

Despite major advances in stroke systems of care, geographic distance continues to represent a critical structural barrier to timely access to reperfusion therapies. Patients residing farther from specialized stroke centers are less likely to receive IVT or MT and are more likely to experience clinically meaningful delays that adversely affect outcomes (7, 8). To mitigate these barriers, hub-and-spoke models have been widely implemented, centralizing advanced stroke care in comprehensive centers while relying on structured referral pathways from peripheral hospitals (9). However, in middle-income countries characterized by complex geography and uneven distribution of healthcare resources, this model may inadvertently reinforce disparities between urban and rural populations.

Beyond geographic distance, several organizational factors within stroke systems of care have been identified as important determinants of treatment timeliness. These include the timely activation and use of emergency medical services, prehospital stroke recognition and pre-notification to receiving hospitals, as well as the availability of coordinated stroke care pathways that facilitate rapid neurological evaluation and therapeutic decision-making. In addition, intra-hospital organizational factors—such as the availability of dedicated stroke units, optimized neuroimaging workflows, and standardized management protocols—can improve the efficiency of acute stroke care. At the system level, strategies such as public education campaigns and telestroke networks have also been shown to contribute to earlier symptom recognition and improved coordination across different levels of care in certain settings (10).

In such settings, additional challenges, including fragmented healthcare delivery, limited prehospital stroke networks, and prolonged interfacility transfer times, further compromise timely access to definitive care (11). Although certified stroke centers routinely monitor in-hospital quality metrics such as door-to-CT, door-to-needle, and door-to-groin puncture times, there remains a critical evidence gap regarding how patients’ geographic origin influences stroke severity at presentation, access to reperfusion therapies within recommended therapeutic windows, and subsequent mortality and functional outcomes (12). Addressing this gap is essential for strengthening regional stroke networks and reducing inequities in access to high-quality neurovascular care. Accordingly, the objective was to evaluate the association between geographic distance to a Comprehensive Stroke Center and clinical outcomes in patients with acute ischemic stroke in a middle-income country.

## 2 Methods

### 2.1 Study design and setting

We conducted a retrospective cohort study using data derived from a structured institutional stroke registry at a certified Center of Excellence for stroke care located in northeastern Colombia. The registry includes patients who met predefined inclusion and exclusion criteria after initial administrative identification. The center operates under standardized clinical pathways and quality indicators aligned with institutional clinical practice guidelines and international recommendations for acute stroke management (13). The registry prospectively captures standardized clinical, functional, and process-of-care data for all patients admitted with stroke.

### 2.2 Eligibility criteria and patient selection

All consecutive adult patients (≥18 years) admitted between November 2024 and January 2026 with a confirmed diagnosis of acute ischemic stroke were eligible for inclusion. Stroke diagnosis was established through clinical evaluation and confirmatory neuroimaging, in accordance with institutional clinical practice guidelines. Potentially eligible cases were initially identified through administrative screening using ICD-10 codes corresponding to acute ischemic stroke (I63 and related subcodes). These cases were subsequently reviewed, and patients meeting the study eligibility criteria were incorporated into the institutional stroke registry. To ensure accurate assessment of geographic exposure, only patients with a clearly documented municipality of origin at the time of stroke and whose index admission occurred within the study period were included.

**Figure 1:**
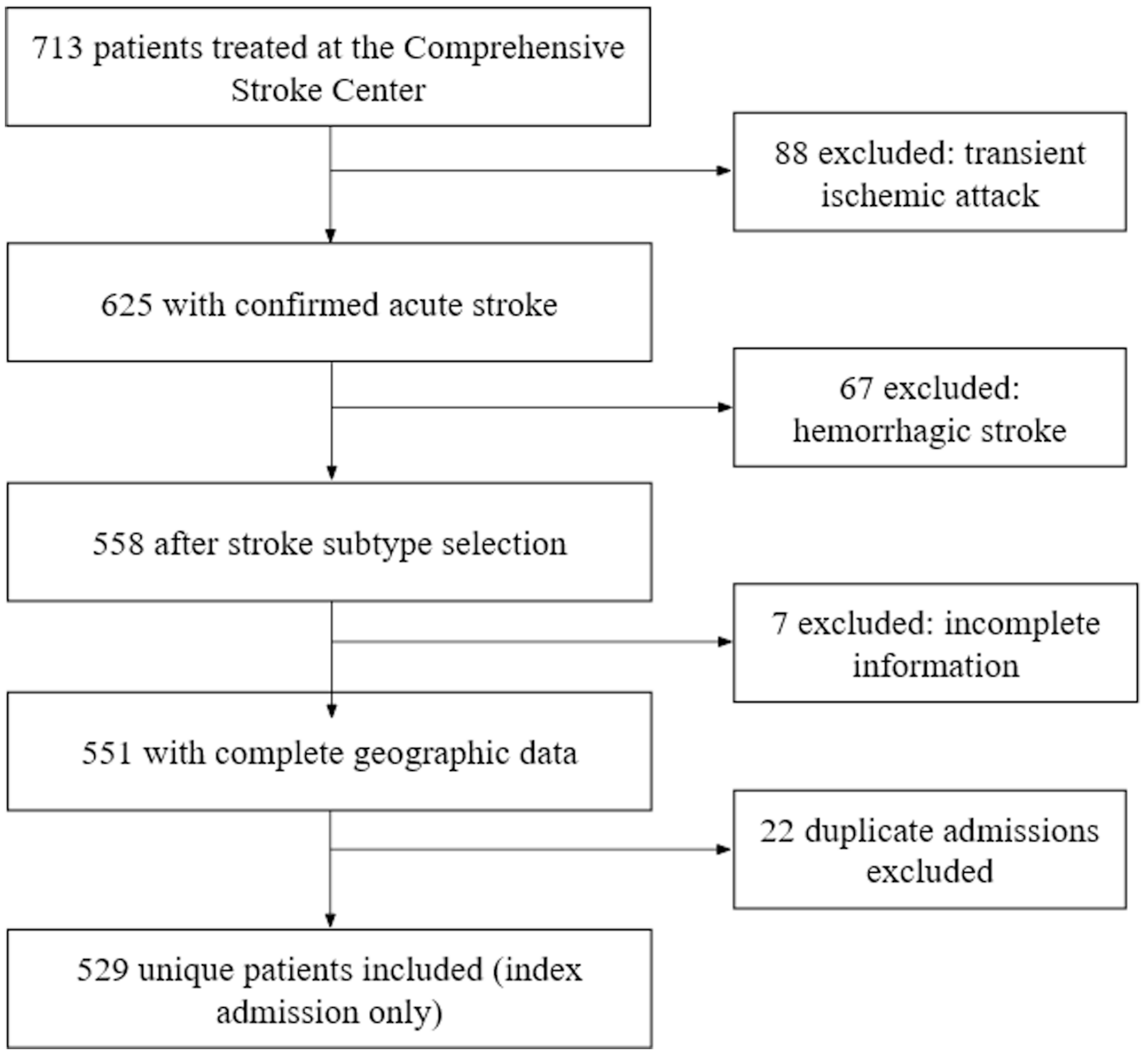
Flow diagram of patient selection for the study cohort. Flow diagram of patient selection. Of 713 patients treated at the Comprehensive Stroke Center, 625 had confirmed acute ischemic stroke. Patients with transient ischemic attack or hemorrhagic stroke were excluded, followed by exclusion of cases with incomplete geographic information and duplicate admissions. The final analytic cohort comprised 529 unique patients.

Patients were excluded if they had transient ischemic attack, intracerebral hemorrhage, subarachnoid hemorrhage, epidural or subdural hematomas, patients with acute neurological deficits secondary to head trauma were excluded. Patients with primary intracerebral hemorrhage were excluded. Hemorrhagic transformation of ischemic stroke was retained as an in-hospital complication when applicable. Records lacking confirmatory neuroimaging, those without sufficient information to establish geographic origin, and duplicate records corresponding to repeated hospitalizations were also excluded. For patients with multiple admissions during the study period, only the index hospitalization was retained to avoid correlated observations. The patient selection process is summarized in Figure 1.

### 2.3 Exposure

The primary exposure of interest was the geographic distance between the patient’s municipality of origin at the time of stroke and the treating Comprehensive Stroke Center. Distance was calculated in kilometers as a continuous variable using Google Maps (14), selecting the fastest available road route. To ensure standardization and reproducibility, all measurements were obtained using identical platform settings and routing parameters.

**Figure 2:**
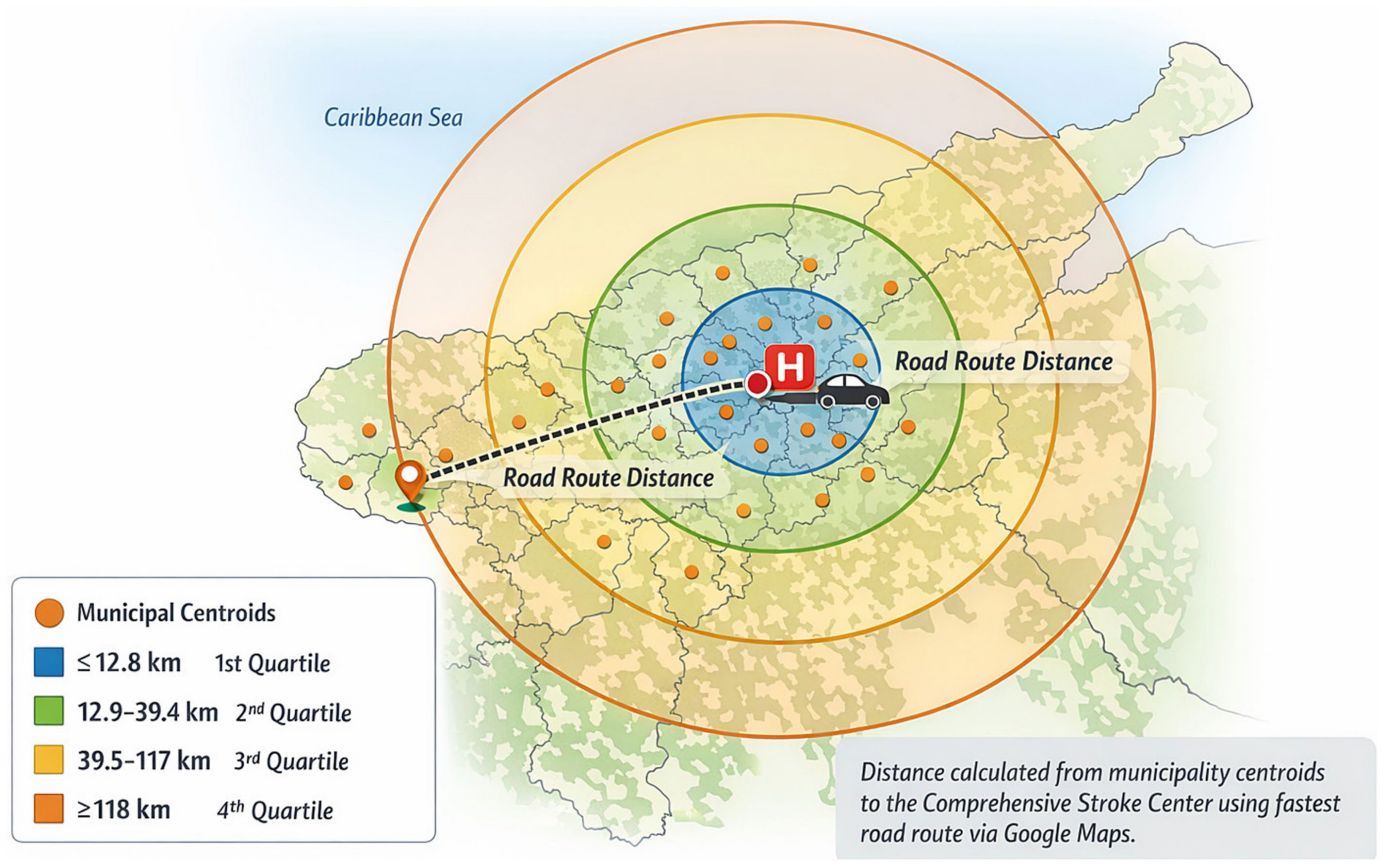
Determination of distance to the Stroke Center. Municipalities of origin were represented by geographic centroids derived from official administrative boundaries provided by the National Administrative Department of Statistics of Colombia (DIVIPOLA). Road-network distance between each municipal centroid and the Comprehensive Stroke Center was calculated using the fastest available road route in Google Maps. Distances were measured in kilometers and analyzed as a continuous variable. For descriptive purposes, distances were additionally categorized into quartiles (≤12.8 km, 12.9–39.4 km, 39.5–117 km, and ≥118 km), represented as concentric distance bands surrounding the treating center.

Geographic distance was defined at the municipal level using centroid-based road routing. For each municipality of origin, geographic centroids derived from official administrative boundaries provided by the National Administrative Department of Statistics of Colombia (DIVIPOLA) were used as representative origin points. Road-network distance between each centroid and the treating center was then calculated. Distance was analyzed as a continuous variable and, for descriptive and visualization purposes, additionally categorized into quartiles (Figure 2).

### 2.4 Baseline characteristics

Baseline sociodemographic and administrative variables included age, sex, health insurance regime, and relevant comorbidities, including arterial hypertension. Administrative aspects of care, such as mode of hospital admission, hospital readmission during the study period, and discharge status were also recorded.

### 2.5 Clinical outcomes and quality indicators

Stroke severity at admission assessed using the National Institutes of Health Stroke Scale (NIHSS) (15), a standardized clinical tool that quantifies stroke-related neurological deficit across multiple domains, including level of consciousness, motor function, sensory loss, language, and visual fields, with higher scores indicating greater severity. Functional outcome at admission and discharge was evaluated using the modified Rankin Scale (mRS) (16), a widely used ordinal scale that measures global disability and dependence in daily activities, ranging from no symptoms to severe disability or death. Adherence to institutional and national stroke quality indicators was documented, including venous thromboembolism prophylaxis, speech and language pathology assessment, rehabilitation services, antithrombotic therapy, anticoagulation for atrial fibrillation, and indicators of intravenous thrombolysis and mechanical thrombectomy. Major in-hospital complications were recorded, including pneumonia, aspiration pneumonia, pulmonary embolism, deep vein thrombosis and urinary tract infection. Additional in-hospital interventions, such as gastrostomy placement and decompressive craniectomy.

### 2.6 Time metrics

Among the process-of-care metrics, the time from symptom onset to hospital arrival, door-to-computed tomography time, door-to-needle time, door-to-groin puncture time and puncture-to-recanalization time. Imaging-based stroke severity was assessed using the Alberta Stroke Program Early CT Score (ASPECTS) (17), a 10-point quantitative score applied to non-contrast brain CT that evaluates the extent of early ischemic changes in the middle cerebral artery territory, where lower scores reflect larger infarct cores. Standard process-of-care time metrics were recorded, including symptom onset-to-door time, door-to-computed tomography time, door-to-needle time for intravenous thrombolysis, door-to-groin puncture time, and puncture-to-recanalization time for mechanical thrombectomy (13).

### 2.7 Statistical analysis

Spatial distribution and distance gradients were visualized using municipal-level choropleth maps for Colombia and, in a sub-analysis, for the Department of Santander. Administrative boundaries were obtained from the Global Administrative Areas (GADM) database. Patient records were geocoded using the centroid coordinates of the municipality of origin, and municipalities were colored according to distance category using a consistent color scale. All spatial preprocessing, distance classification, and visualization were performed in R using the packages *sf*, *geodata*, *terra*, *dplyr*, *ggplot2*, and *ggspatial*.

Continuous variables were assessed for normality using the Shapiro–Wilk test. Normally distributed variables are presented as means with standard deviations, and non-normally distributed variables as medians with interquartile ranges. Categorical variables are summarized as frequencies and percentages.

For bivariate comparisons, chi-square tests were used for categorical variables and the Kruskal–Wallis test for non-normally distributed continuous variables. Functional outcome at discharge was assessed using the mRS; primary analyses employed ordinal logistic regression under the proportional odds assumption. In-hospital case-fatality was analyzed using multivariable logistic regression. Patients with missing discharge mRS were excluded from the ordinal regression analyses. The model was constructed using candidate covariates selected *a priori* based on clinical relevance and prior evidence; variables with a *p* value < 0.25 in univariable analyses were considered for inclusion. A backward elimination strategy was applied, retaining variables that were statistically significant or clinically relevant. Multicollinearity was assessed using variance inflation factors. For logistic regression models, calibration was evaluated using the Hosmer–Lemeshow goodness-of-fit test and discrimination using the area under the receiver operating characteristic curve. Results are reported as odds ratios (OR) with 95% confidence intervals (CI). For ordinal models, overall model fit was assessed using the likelihood ratio test, and the proportional odds assumption was evaluated using the Brant test. Statistical significance was defined as a two-sided *p* value < 0.05. All analyses were conducted using R and Stata version 16.

### 2.8 Ethical statement

The study was approved by the institutional ethics committee (Code: CEI-2024-0746-33). In accordance with the Colombian Ministry of Health regulations, the study was classified as minimal risk. The research was conducted in full compliance with national and international ethical standards, including the principles of the Declaration of Helsinki, the Belmont Report, and the Council for International Organizations of Medical Sciences (CIOMS) guidelines. Personal data were handled in strict accordance with Colombian data protection legislation (Law 1581 of 2012 and its regulatory decrees). All data were anonymized prior to analysis to ensure confidentiality and the protection of participants’ privacy.

## 3 Results

A total of 529 patients with acute ischemic stroke were included in the analysis. The geographic origin of patients showed a highly centralized spatial pattern, with a progressive decrease in case frequency as distance from the health institution increased. As illustrated in the map, municipalities located in close proximity to the institution accounted for the largest proportion of patients, whereas municipalities located at greater distances were more sparsely distributed across the national territory.

For descriptive analyses, geographic distance was categorized into quartiles based on the distribution of the study population: ≤12.8 km, 12.9–39.4 km, 39.5–117 km, and ≥118 km. Overall, 215 patients (40.64%) resided in municipalities located ≤12.8 km from the health institution, largely corresponding to the immediate metropolitan area. An additional 52 patients (9.83%) originated from municipalities situated between 12.9 and 39.4 km, representing a second zone of regional influence.

**Figure 3:**
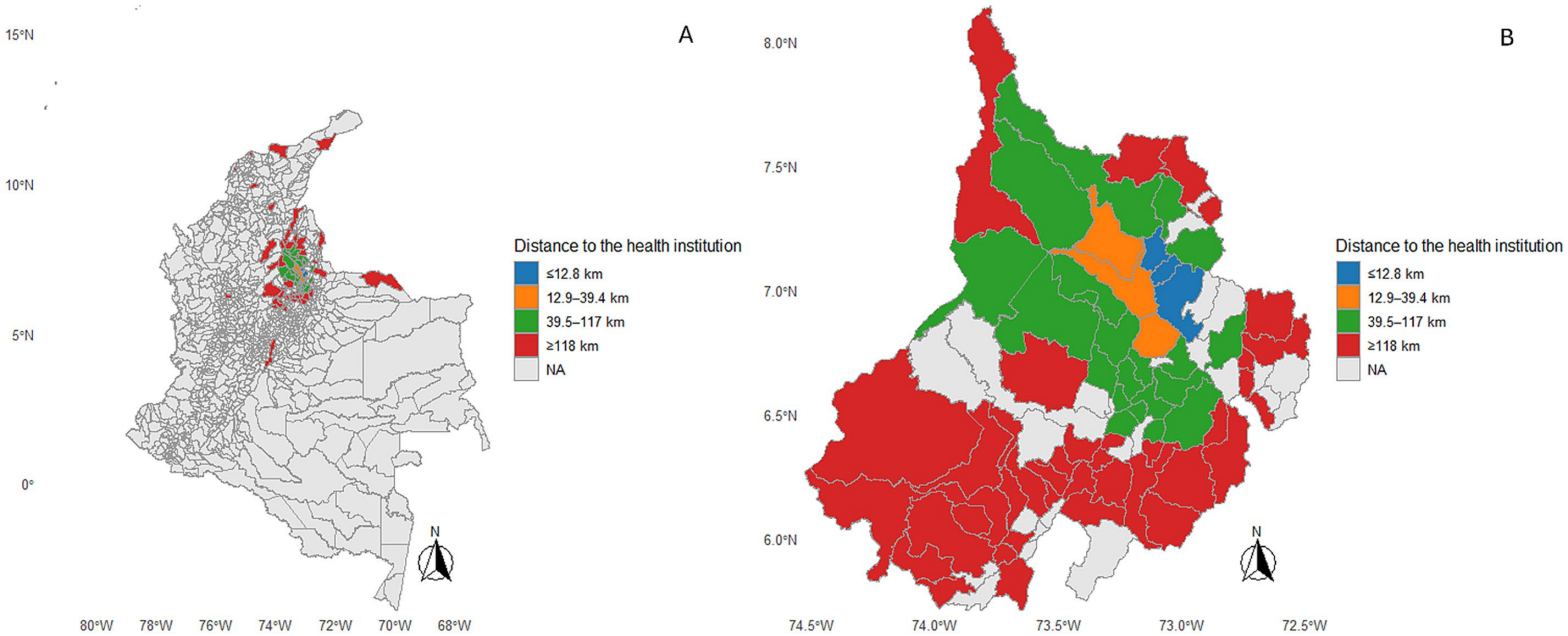
Geographic distribution of patients with acute ischemic stroke in the country **(A)** and the Department of Santander **(B)** according to distance from the treating center**. (A)** Colombia. **(B)** Department of Santander. Municipalities are colored according to road distance from the municipal centroid to the treating center: ≤12.8 km (blue), 12.9–39.4 km (orange), 39.5–117 km (green), and ≥118 km (red). Grey indicates municipalities with no registered cases.

Patients whose place of origin at the time of stroke onset was located between 39.5 and 117 km accounted for 131 cases (24.76%), indicating substantial referral from intermediate regional areas. A comparable proportion of patients (131 cases; 24.76%) originated from municipalities located at distances of ≥118 km, including both peripheral municipalities within the department and municipalities from other regions of the country. This group reflects referrals from geographically distant areas to the health institution (Figure 3).

In this study, overall, patients were predominantly older adults, with median age values across distance categories ranging from 71.0 to 75.0 years, without significant differences between groups (*p* = 0.372). Male sex predominated across all distance categories, accounting for 57.69–61.86% of the cohort, with no significant variation by geographic distance (*p* = 0.885). Clear differences were observed in the health system–related variables. As geographic distance from the referral center increased, there was a progressive rise in affiliation with the subsidized insurance regime (from 51.16% in the closest group to 87.02% in the most distant group; *p* < 0.001) (Table 1).

**Table 1:** Baseline demographic and clinical characteristics according to geographic distance categories.

Variable	Categories	≤12.8 km *n* (%) 215 (40.64)	12.9–39.4 km *n* (%) 52 (9.84)	39.5–117 km *n* (%) 131 (24.76)	≥118 km *n* (%) 131 (24.76)	*p*-value
Age, years	Median (IQR)	71.00 (59.00–81.00)	71.50 (59.50–81.00)	75.00 (64.00–82.00)	73.00 (63.00–80.00)	0.372^†^
Sex	Female	82 (38.14)	22 (42.31)	53 (40.46)	55 (41.98)	0.885
	Male	133 (61.86)	30 (57.69)	78 (59.54)	76 (58.02)	
Health insurance regime	Subsidized	110 (51.16)	40 (76.92)	105 (80.15)	114 (87.02)	<0.001
	Contributory	79 (36.74)	10 (19.23)	16 (12.21)	10 (7.63)	
	Other	26 (12.09)	2 (3.85)	10 (7.63)	7 (5.34)	
Arterial hypertension	Yes	132 (61.40)	38 (73.08)	93 (70.99)	78 (59.54)	0.094
Mode of arrival	Ambulance	11 (5.12)	0 (0.00)	1 (0.76)	1 (0.76)	<0.001
	In-hospital onset/already hospitalized	5 (2.33)	0 (0.00)	2 (1.53)	1 (0.76)	
	Self-transport (own means)	89 (41.40)	19 (36.54)	19 (14.50)	17 (12.98)	
	Referred (interfacility transfer)	110 (51.16)	33 (63.46)	109 (83.21)	112 (85.50)	
Readmission	Yes	1 (0.47)	0 (0.00)	1 (0.76)	0 (0.00)	0.740
Discharge status	Alive	194 (90.23)	44 (84.62)	118 (90.08)	122 (93.13)	0.371
	Death	21 (9.77)	8 (15.38)	13 (9.92)	9 (6.87)	

Age is reported as median and interquartile range (IQR). Distance categories were defined according to percentiles of the distribution of geographic distance (km) from the patient’s municipality of residence to the Comprehensive Stroke Center. *p* values were calculated using the Pearson chi-square test for categorical variables and the Kruskal–Wallis test for continuous variables (†). A two-sided *p* value <0.05 was considered statistically significant.

Hypertension was highly prevalent across all groups (59.54–73.08%) and did not differ significantly by distance (*p* = 0.094). However, patterns of hospital access varied according to geographic proximity. Patients residing closer to the referral center more frequently arrived by their own means (41.40% vs. 12.98% in the most distant group), whereas interfacility referral predominated among patients from more distant municipalities, increasing from 51.16% in the closest category to 85.50% in the farthest (*p* < 0.001). Direct ambulance arrival was uncommon across all distance categories (5.12%). Readmissions during the study period were rare (0.76%) and showed no meaningful variation across geographic categories (*p* = 0.740) (Table 1).

Stroke severity at presentation was similar across distance categories, with most patients presenting with minor to moderate ischemic stroke. Severe ischemic stroke accounted for 10.70–20.61% of cases across categories (*p* = 0.327). Functional status at admission did not differ significantly by distance, with 47.33–57.67% of patients presenting with a mRS score of completely independent (*p* = 0.654). At discharge, a higher proportion of patients from more distant municipalities had moderate-to-severe disability (mRS 4-5: 16.74–34.35%), compared with those residing closer to the center, and this difference reached statistical significance (*p* = 0.041) (Table 2).

**Table 2:** Stroke-related characteristics, quality metrics, and in-hospital complications by geographic distance category.

Variable	Categories	≤12.8 km *n* (%)	12.9–39.4 km *n* (%)	39.5–117 km *n* (%)	≥118 km *n* (%)	*p* (*χ*^2^)
Ischemic stroke severity category	Minor (NIHSS 0–4)	75 (34.88)	16 (30.77)	40 (30.53)	36 (27.48)	0.327
	Moderate (NIHSS 5–15)	92 (42.79)	22 (42.31)	62 (47.33)	57 (43.51)	
	Moderate to severe (NIHSS 16–20)	25 (11.63)	8 (15.38)	12 (9.16)	11 (8.40)	
	Severe (NIHSS 21–42)	23 (10.70)	6 (11.54)	17 (12.98)	27 (20.61)	
mRS at admission	Completely independent	124 (57.67)	28 (53.85)	62 (47.33)	66 (50.38)	0.654
	No significant disability	37 (17.21)	14 (26.92)	30 (22.90)	26 (19.85)	
	Slight disability	18 (8.37)	2 (3.85)	14 (10.69)	13 (9.92)	
	Moderate disability	21 (9.77)	6 (11.54)	13 (9.92)	12 (9.16)	
	Moderately severe disability	7 (3.26)	1 (1.92)	9 (6.87)	7 (5.34)	
	Severe disability	5 (2.33)	0 (0.00)	3 (2.29)	3 (2.29)	
	NA	1 (0.47)	0 (0.00)	0 (0.00)	0 (0.00)	
	No record	2 (0.93)	1 (1.92)	0 (0.00)	4 (3.05)	
mRS at discharge	Completely independent	23 (10.70)	6 (11.54)	10 (7.63)	6 (4.58)	0.041
	No significant disability	33 (15.35)	9 (17.31)	21 (16.03)	24 (18.32)	
	Slight disability	40 (18.60)	6 (11.54)	21 (16.03)	9 (6.87)	
	Moderate disability	26 (12.09)	7 (13.46)	20 (15.27)	19 (14.50)	
	Moderately severe	32 (14.88)	7 (13.46)	22 (16.79)	34 (25.95)	
	Severe disability	4 (1.86)	3 (5.77)	10 (7.63)	11 (8.40)	
	Death	19 (8.84)	7 (13.46)	13 (9.92)	9 (6.87)	
	No record	38 (17.67)	7 (13.46)	14 (10.69)	19 (14.50)	
VTE prophylaxis	Yes	154 (71.63)	34 (65.38)	102 (77.86)	104 (79.39)	0.042
Speech-language pathology	Yes	185 (86.05)	46 (88.46)	117 (89.31)	113 (86.26)	0.639
Rehabilitation	Yes	200 (93.02)	50 (96.15)	121 (92.37)	124 (94.66)	0.865
Antithrombotic therapy	Yes	134 (62.33)	34 (65.38)	75 (57.25)	76 (58.02)	0.632
Anticoagulation for AF	Yes	57 (26.51)	14 (26.92)	36 (27.48)	39 (29.77)	0.930
Intravenous thrombolysis	Yes	172 (80.00)	41 (78.85)	112 (85.50)	122 (93.13)	0.007
Mechanical thrombectomy	Yes	171 (79.53)	38 (73.08)	116 (88.55)	118 (90.08)	0.004
Major in-hospital complication	Yes	13 (6.05)	6 (11.54)	15 (11.45)	9 (6.87)	0.234
Pneumonia	Yes	4 (1.86)	3 (5.77)	6 (4.58)	6 (4.58)	0.346
Aspiration pneumonia	Yes	3 (1.40)	0 (0.00)	2 (1.53)	1 (0.76)	0.785
Pulmonary embolism	Yes	0 (0.00)	2 (3.85)	0 (0.00)	0 (0.00)	<0.001
Deep vein thrombosis	Yes	1 (0.47)	0 (0.00)	0 (0.00)	0 (0.00)	0.691
Urinary tract infection	Yes	2 (0.93)	0 (0.00)	3 (2.29)	2 (1.53)	0.587
Other complications	Yes	4 (1.86)	2 (3.85)	4 (3.05)	0 (0.00)	0.208
Gastrostomy during hospitalization	Yes	8 (3.72)	4 (7.69)	8 (6.11)	13 (9.92)	0.135
Decompressive craniectomy	Yes	2 (0.93)	0 (0.00)	2 (1.53)	2 (1.53)	0.792

Data are shown as *n* (%). Distance categories were defined by percentiles (km). Neurological status was assessed using the National Institutes of Health Stroke Scale (NIHSS) and functional status the modified Rankin Scale (mRS). *χ*^2^ test was used for categorical comparisons.

Adherence to stroke quality indicators was high across distance categories. Venous thromboembolism prophylaxis showed modest variation (65.38–79.39%; *p* = 0.042), whereas speech-language pathology assessment (86.05–89.31%), rehabilitation services (92.37–96.15%), antithrombotic therapy (57.25–65.38%), and anticoagulation for atrial fibrillation (26.51–29.77%) did not differ significantly by geographic distance. In contrast, the use of reperfusion therapies increased with distance, with intravenous thrombolysis ranging from 78.85 to 93.13% (*p* = 0.007) and mechanical thrombectomy from 73.08 to 90.08% (*p* = 0.004). Major in-hospital complications were uncommon, occurring in 6.05–11.54% of patients across categories (*p* = 0.234). Pulmonary embolism was rare but differed across categories, with events observed exclusively in the 12.9–39.4 km category (3.85%) (*p* < 0.001). Gastrostomy during hospitalization ranged from 3.72 to 9.92%, without statistically significant differences by distance (*p* = 0.135) (Table 2).

Prehospital time from symptom onset to hospital arrival tended to increase with greater geographic distance, without reaching statistical significance. In contrast, intrahospital time metrics (door-to-CT, door-to-needle, door-to-groin, and puncture-to-recanalization) and ASPECTS scores were comparable across all distance categories, indicating similar in-hospital performance regardless of patient origin (Table 3).

**Table 3:** Outcomes related to in-hospital times according to geographic distance categories.

Variable	≤12.8 km	12.9–39.4 km	39.5–117 km	≥118 km	*p* value
Time from symptom onset to hospital arrival, min	139.5 (55.5–252.5)	193 (141–353)	241 (91–407)	252 (107–465)	0.082
Door-to-CT time	12 (7–26)	18 (13–40)	15 (8–21)	26 (12–37)	0.368
Door-to-needle time	48 (32–58.5)	27.5 (20.5–59)	41 (29–59)	40 (38–54)	0.728
Door-to-groin puncture time	77 (19–92)	53 (22–54)	79.5 (28–149)	45 (2–88)	0.578
Puncture-to-recanalization time	33 (25–53)	22.5 (12–35)	21 (20–64)	35 (35–35)	0.584
ASPECTS	9 (7–10)	9 (8–10)	8.5 (7–10)	9 (5–10)	0.933

Data are presented as median (interquartile range). Times are in minutes. ASPECTS indicates Alberta Stroke Program Early CT Score. P values reflect comparisons across geographic distance categories.

**Figure 4:**
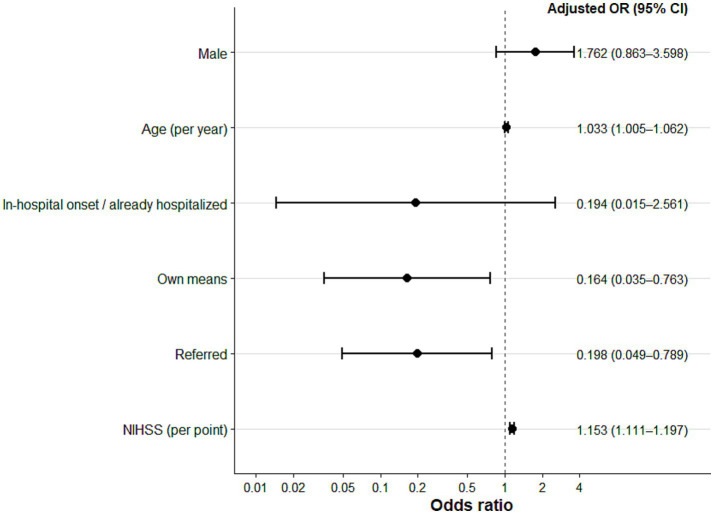
Forest plot of factors independently associated with in-hospital case-fatality. *Adjusted for sex (ref. female), age, mode of arrival (ref. ambulance), and NIHSS.

In multivariable logistic regression, older age and greater neurological severity at admission were independently associated with higher in-hospital case-fatality. Each additional year of age increased the possibility of death by approximately 3% (OR 1.03; 95% CI 1.00–1.06), while each one-point increase in NIHSS was associated with a 15% higher possibility of mortality (OR 1.15; 95% CI 1.11–1.197). Mode of arrival was also associated with the outcome, as patients arriving by their own means or referred from another institution had lower odds of in-hospital death compared with the reference group. Sex was not independently associated with mortality. The model showed adequate calibration (Hosmer–Lemeshow *p* = 0.136) and strong discrimination (AUC = 0.85) (Figure 4).

In the ordered logistic regression model, higher ln (KM) values and older age were independently associated with worse functional outcomes at hospital discharge, as measured by the mRS. Each one-unit increase in ln (KM) was associated with a 25% higher odds of being in a worse mRS category (OR = 1.25; 95% CI: 1.09–1.44), while each additional year of age increased the odds of greater disability by approximately 3% (OR = 1.03; 95% CI: 1.02–1.04). Model fit was assessed using the likelihood ratio test. The ordered logistic regression model showed a statistically significant overall fit (LR *χ*^2^ = 36.18; *p* < 0.001). The proportional odds assumption was evaluated using the Brant test and was not violated.

## 4 Discussion

In this study, we evaluated the relationship between geographic distance and clinical and functional outcomes, as well as processes of care, in patients with acute ischemic stroke treated at a Comprehensive Stroke Center. Geographic distance was not independently associated with in-hospital case-fatality. Instead, multivariable modeling showed that early case-fatality risk was primarily explained by age and stroke severity at admission (13, 18, 19). However, geographic distance and age were independently associated with worse functional outcomes at hospital discharge.

From a sociodemographic perspective, clear structural differences were observed across geographic distance categories. A higher proportion of patients affiliated with the subsidized health insurance regime was identified among those originating from more distant municipalities. This distribution is consistent with the territorial organization of the Colombian health system and with prior reports from Latin America describing a greater concentration of socially vulnerable populations in rural and peripheral areas (20, 21). Additionally, the low frequency of direct ambulance transport highlights persistent limitations in prehospital emergency medical services, a factor widely recognized as a determinant of timely access to reperfusion therapies (22, 23).

In this context, geographic distance may also act as a proxy for broader social determinants of health. Patients residing in more remote municipalities are more likely to experience socioeconomic disadvantage, reduced access to specialized healthcare services, and structural barriers within the health system. These conditions may independently increase vulnerability to poorer functional outcomes following stroke, beyond the direct effect of physical distance to the treating center (24).

Regarding clinical characteristics, stroke severity at admission and baseline functional status were comparable across distance categories, suggesting that patients accessed the referral center with similar initial clinical profiles. Increasing age and higher admission NIHSS were independently associated with higher in-hospital case-fatality. These findings are consistent with prior evidence identifying advanced age and initial stroke severity as the most robust predictors of early mortality following acute ischemic stroke (25). Observational studies and analyses from large international registries have demonstrated that these factors reflect not only a greater extent of acute cerebral injury but also reduced physiological reserve and increased vulnerability to systemic complications during hospitalization (26). These findings reinforce the external validity of previously established determinants of stroke outcomes. Even within regionalized stroke care systems with standardized intrahospital processes, age and baseline neurological severity remain key drivers of early case-fatality risk in acute ischemic stroke (12, 27–29). In this context, the higher likelihood of favorable discharge outcomes observed among referred patients in the adjusted model likely reflects referral-related selection rather than a true advantage conferred by transfer itself.

Despite similar clinical profiles at admission, a higher proportion of moderate-to-severe disability at hospital discharge was observed among patients originating from more distant municipalities. In the adjusted ordinal model, geographic distance remained independently associated with worse functional outcomes at discharge, alongside age (5, 7, 27). The adequate fit of the regression model supports the robustness of these associations across the spectrum of functional outcomes measured by the modified Rankin Scale. This finding aligns with previous evidence suggesting that greater geographic distance may adversely affect functional recovery through prehospital delays and structural barriers rather than deficiencies in intrahospital care delivery (30). Therefore, this association should be interpreted cautiously, as unmeasured prehospital factors such as longer onset-to-door times, variability in initial management at referring hospitals, and differences in post-discharge support may contribute to functional outcomes beyond the effect of distance alone.

An important finding of the study is the consistency observed in intrahospital processes of care. Indicators related to treatment times, venous thromboembolism prophylaxis, speech and language pathology evaluation, early rehabilitation, and antithrombotic therapy showed high adherence across all distance categories. These results suggest that once patients reach the Comprehensive Stroke Center, care delivery is homogeneous and aligned with international recommendations, regardless of geographic origin (6, 31, 32). However, the study design does not allow precise evaluation of prehospital delays, which remain a critical component of the stroke care continuum.

This study has limitations inherent to its observational design and the institutional nature of the registry. Referral and selection bias are possible, as this high-complexity center receives patients from a wide geographic area and includes only those who reached and were admitted, potentially underrepresenting individuals with limited access to care. Although data completeness was high, residual missing data bias may be present. Finally, functional outcomes were assessed using the modified Rankin Scale at hospital discharge, reflecting short-term status without capturing long-term recovery.

Overall, these results indicate that geographic distance was not associated with differences in intrahospital quality-of-care metrics or in-hospital case-fatality. However, greater distance remained independently associated with worse functional status at discharge, highlighting the importance of strengthening prehospital stroke systems and post-acute rehabilitation pathways for patients from geographically remote areas (33).

## 5 Conclusion

Within a regionalized stroke network, geographic distance to the Comprehensive Stroke Center was not independently associated with in-hospital case-fatality or with intrahospital quality-of-care metrics, which were largely explained by patient-level clinical factors such as age and admission stroke severity. However, greater geographic distance remained independently associated with worse functional status at hospital discharge. These findings suggest that inequities linked to geography likely operate through prehospital and post-acute pathways rather than through differences in in-hospital care, underscoring the need to strengthen emergency medical transport, interfacility transfer processes, and access to timely post-stroke rehabilitation for patients from remote areas.

## Data Availability

The raw data supporting the conclusions of this article will be made available by the authors, without undue reservation.

## References

[1] Ren H Liu Y Zhao M Shen H Nie S Gao X . Stroke: epidemiology, risk factors, signaling pathways, and clinical management. MedComm. (2025) 6:e70558. doi: 10.1002/mco2.70558, 41427019 PMC12711381

[2] GBD 2019 Stroke Collaborators. Global, regional, and national burden of stroke and its risk factors, 1990–2019: a systematic analysis for the global burden of disease study 2019. Lancet Neurol. (2021) 20:795–820. doi: 10.1016/S1474-4422(21)00252-0 34487721 PMC8443449

[3] Emberson J Lees KR Lyden P Blackwell L Albers G Bluhmki E . Effect of treatment delay, age, and stroke severity on the effects of intravenous thrombolysis with alteplase for acute ischaemic stroke: a meta-analysis of individual patient data from randomised trials. Lancet. (2014) 384:1929–35. doi: 10.1016/S0140-6736(14)60584-5, 25106063 PMC4441266

[4] Goyal M Demchuk AM Menon BK Eesa M Rempel JL Thornton J . Randomized assessment of rapid endovascular treatment of ischemic stroke. N Engl J Med. (2015) 372:1019–30. doi: 10.1056/NEJMoa1414905, 25671798

[5] Saver JL. Time is brain—quantified. Stroke. (2006) 37:263–6. doi: 10.1161/01.STR.0000196957.55928.ab, 16339467

[6] Saver JL Goyal M van der Lugt A Menon BK Majoie CBLM Dippel DWJ . Time to treatment with endovascular thrombectomy and outcomes from ischemic stroke: a meta-analysis. JAMA. (2016) 316:1279–88. doi: 10.1001/jama.2016.13647 27673305

[7] Adeoye O Albright KC Carr BG Wolff C Mullen MT Abruzzo T . Geographic access to acute stroke care in the United States. Stroke. (2014) 45:3019–24. doi: 10.1161/STROKEAHA.114.006293, 25158773 PMC5877807

[8] Froehler MT Saver JL Zaidat OO Jahan R Aziz-Sultan MA Klucznik RP . Interhospital transfer before thrombectomy is associated with delayed treatment and worse outcome in the STRATIS registry. Circulation. (2017) 136:2311–21. 28943516 10.1161/CIRCULATIONAHA.117.028920 PMC5732640

[9] Adeoye O Nyström KV Yavagal DR Luciano J Nogueira RG Zorowitz RD . Recommendations for the establishment of stroke systems of care: a 2019 update. Stroke. (2019) 50:e187–210. doi: 10.1161/str.0000000000000173, 31104615

[10] Botelho A Rios J Fidalgo AP Ferreira E Nzwalo H. Organizational factors determining access to reperfusion therapies in ischemic stroke: systematic literature review. Int J Environ Res Public Health. (2022) 19:16357. doi: 10.3390/ijerph192316357, 36498429 PMC9735885

[11] Bosch J Lotlikar R Melifonwu R Roushdy T Sebastian I Abraham SV . Prehospital stroke care in low- and middle-income countries: a world stroke organization scientific statement. Int J Stroke. (2025) 20:918–27. doi: 10.1177/17474930251351867 40495741 PMC12446713

[12] Temel MH Erden Y Bağcıer F. Rural-urban disparities in stroke outcomes: unveiling quality of life, self-efficacy and healthcare utilization patterns of stroke patients in Türkiye. Rural Remote Health. (2025) 25:9477. doi: 10.22605/RRH9477, 40857757

[13] Powers WJ Rabinstein AA Ackerson T Adeoye OM Bambakidis NC Becker K . Guidelines for the early management of patients with acute ischemic stroke: 2019 update to the 2018 guidelines. Stroke. (2019) 50:e344–418. doi: 10.1161/STR.0000000000000211 31662037

[14] Google. Google Maps. Mountain View, CA: Google LLC.

[15] Brott T Adams HP Olinger CP Marler JR Barsan WG Biller J . Measurements of acute cerebral infarction: a clinical examination scale. Stroke. (1989) 20:864–70. doi: 10.1161/01.str.20.7.864, 2749846

[16] van Swieten JC Koudstaal PJ Visser MC Schouten HJ van Gijn J. Interobserver agreement for the assessment of handicap in stroke patients. Stroke. (1988) 19:604–7. doi: 10.1161/01.STR.19.5.604, 3363593

[17] Barber PA Demchuk AM Zhang J Buchan AM. Validity and reliability of a quantitative computed tomography score in predicting outcome of hyperacute stroke before thrombolytic therapy. Lancet. (2000) 355:1670–4. doi: 10.1016/S0140-6736(00)02237-6, 10905241

[18] Goyal M Menon BK van Zwam WH Dippel DWJ Mitchell PJ Demchuk AM . Endovascular thrombectomy after large-vessel ischaemic stroke: a meta-analysis of individual patient data from five randomised trials. Lancet. (2016) 387:1723–31. doi: 10.1016/S0140-6736(16)00163-X 26898852

[19] Lindsay P Furie KL Davis SM Donnan GA Norrving B. World stroke organization global stroke services guidelines and action plan. Int J Stroke. (2014) 9:4–13. doi: 10.1111/ijs.12371, 25250836

[20] Venema E Groot AE Lingsma HF Hinsenveld W Treurniet KM Chalos V . Effect of interhospital transfer on endovascular treatment for acute ischemic stroke. Stroke. (2019) 50:923–30. doi: 10.1161/STROKEAHA.118.024091, 30862265 PMC6430601

[21] Guisado-Alonso D Martínez-Domeño A Prats-Sánchez L Delgado-Mederos R Camps-Renom P Abilleira S . Reasons for not performing mechanical thrombectomy: a population-based study of stroke codes. Stroke. (2021) 52:2746–53. doi: 10.1161/STROKEAHA.120.032648, 34289711

[22] Leira EC Russman AN Biller J Brown DL Bushnell CD Caso V . Preserving stroke care during the COVID-19 pandemic: potential issues and solutions. Neurology. (2020) 95:124–33. doi: 10.1212/WNL.0000000000009713, 32385186 PMC7455350

[23] Yaghi S Willey JZ Cucchiara B Goldstein JN Gonzales NR Khatri P . Treatment and outcome of hemorrhagic transformation after intravenous alteplase in acute ischemic stroke: a scientific statement. Stroke. (2017) 48:e343–61. 29097489 10.1161/STR.0000000000000152

[24] Nguyen MTH Sakamoto Y Maeda T Woodward M Anderson CS Catiwa J . Influence of socioeconomic status on functional outcomes after stroke: a systematic review and meta-analysis. medRxiv. (2023) 13:e033078 10.1161/JAHA.123.033078 PMC11179939 38639361

[25] Adams HP Jr Adams RJ Brott T del Zoppo GJ Furlan A Goldstein LB . Guidelines for the early management of patients with ischemic stroke. Stroke. (2003) 34:1056–83. doi: 10.1161/01.str.0000064841.47697.22, 12677087

[26] Nedeltchev K Renz N Karameshev A Haefeli T Brekenfeld C Meier N . Predictors of early mortality after acute ischaemic stroke. Swiss Med Wkly. (2010) 140:254–9. doi: 10.4414/smw.2010.12919, 20104376

[27] Prabhakaran S Ward E John S Lopes DK Chen M Temes RE . Transfer delay is a major factor limiting the use of intra-arterial treatment in acute ischemic stroke. Stroke. (2011) 42:1626–30. doi: 10.1161/STROKEAHA.110.609750, 21527756

[28] Fischer U Kaesmacher J Mendes Pereira V Chapot R Siddiqui AH Froehler MT . Direct mechanical thrombectomy versus combined intravenous and mechanical thrombectomy in large-artery anterior circulation stroke: a topical review. Stroke. (2017) 48:2912–8. doi: 10.1161/STROKEAHA.117.017208 28887391

[29] Schlemm L Endres M Werring DJ Nolte CH. Benefit of intravenous thrombolysis in acute ischemic stroke patients with high cerebral microbleed burden. Stroke. (2020) 51:232–9. doi: 10.1161/STROKEAHA.119.027633, 31739772

[30] Meretoja A Strbian D Mustanoja S Tatlisumak T Lindsberg PJ Kaste M. Reducing in-hospital delay to 20 minutes in stroke thrombolysis. Neurology. (2012) 79:306–13. doi: 10.1212/WNL.0b013e31825d6011 22622858

[31] Campbell BCV Mitchell PJ Kleinig TJ Dewey HM Churilov L Yassi N . Endovascular therapy for ischemic stroke with perfusion-imaging selection. N Engl J Med. (2015) 372:1009–18. doi: 10.1056/NEJMoa1414792 25671797

[32] Mokin M Primiani CT Siddiqui AH Turk AS. ASPECTS measurement using Hounsfield unit values when selecting patients for stroke thrombectomy. Stroke. (2017) 48:1574–9. doi: 10.1161/STROKEAHA.117.016745, 28487329

[33] GBD 2021 Causes of Death Collaborators. Global burden of 288 causes of death and life expectancy decomposition in 204 countries and territories and 811 subnational locations, 1990–2021: a systematic analysis. Lancet. (2024) 403:2100–32. doi: 10.1016/S0140-6736(24)00367-2 38582094 PMC11126520

